# Heterogeneity-aware risk-adaptive trajectory planning for UAVs in air-ground multi-agent interaction

**DOI:** 10.3389/frobt.2026.1920106

**Published:** 2026-10-05

**Authors:** Ying Wang, Jingwei Wei, Shuai Sun, Erjian Liu

**Affiliations:** 1 School of Traffic Management, People’s Public Security University of China, Beijing, China; 2 The MOT Key Laboratory of Transport Industry of Big Data Application Technologies for Comprehensive Transport, Beijing Jiaotong University, Beijing, China; 3 Department of Computer Science and Technology, Tsinghua University, Beijing, China; 4 Instituto de Física Interdisciplinar y Sistemas Complejos IFISC (CSIC-UIB), Palma deMallorca, Spain

**Keywords:** fuzzy inference, heterogeneous behavior modeling, machine learning for robotics, multi-agent interaction, UAV trajectory planning

## Abstract

Autonomous UAVs sharing low-altitude airspace with ground traffic must continuously interpret the heterogeneous and dynamically varying behaviors of ground agents, a challenge that conventional risk models, largely designed for static obstacles or aircraft-to-aircraft conflicts, are not equipped to address. This study formulates the air-ground interaction problem as a heterogeneous multi-agent risk perception task and proposes a three-stage computational intelligence framework for risk-adaptive UAV trajectory planning. First, a GMM-LightGBM model performs online identification of ground agent driving styles, which is coupled with a Full Velocity Difference (FVD) model to generate personalized agent trajectory predictions. Second, a fuzzy inference system fuses macroscopic traffic context, agent behavioral features, and air-ground geometric relationships into an interpretable, real-time risk score. Third, an MS-DGST-VNet model predicts UAV maneuver parameters, and a risk-adaptive cost function dynamically reweights safety, efficiency, and altitude constraints to generate adaptive trajectories. The proposed driving style classifier achieves 0.96 identification accuracy, and the resulting risk score correlates strongly with three-dimensional air-ground time-to-collision, supporting the framework’s validity. Case studies across representative risk scenarios further demonstrate the framework’s adaptive decision-making capability. By combining machine learning-based behavior identification, fuzzy reasoning, and learning-based trajectory generation, this work contributes a computational intelligence methodology for autonomous UAV decision-making in heterogeneous multi-agent environments.

## 1 Introduction

As urban low-altitude airspace is gradually opened and low-altitude mobility modes such as electric vertical take-off and landing (eVTOL) aircraft and unmanned aerial vehicle (UAV) delivery are deployed at scale, autonomous UAVs are increasingly required to operate as intelligent agents embedded in a shared, dynamic air-ground environment (Liao et al., 2023; Huang et al., 2024; Zhang H. et al., 2023). Within this environment, a UAV must perceive the behavior of the ground agents beneath it in real time, infer the resulting air-ground interaction risk, and adapt its flight decisions accordingly, so as to achieve safe and efficient autonomous operation (Theocharides et al., 2026; Braßel et al., 2025; Gong et al., 2026). However, ground agents, including multiple types of human-driven vehicles, exhibit pronounced behavioral heterogeneity in motion characteristics, risk preferences, and control styles, posing severe challenges to the perception, prediction, and risk quantification capabilities that an autonomous UAV must rely on (Corbetta et al., 2019). As the density of low-altitude operations continues to rise, achieving real-time perception, dynamic risk quantification, and safety-adaptive planning under such behavioral heterogeneity has become a key bottleneck for the autonomous operation of UAVs in air-ground multi-agent environments.

In air-ground multi-agent interaction scenarios, behavioral heterogeneity is a multi-dimensional problem that arises on both sides of the interaction. UAVs themselves exhibit markedly different flight styles depending on mission type, such as logistics delivery, infrastructure inspection, or emergency rescue, and control characteristics (Hu et al., 2026; Dong et al., 2025; Chen et al., 2025). Meanwhile, the human-driven ground agents below also display heterogeneous driving styles, including conservative, standard, and aggressive patterns, and this dynamic behavior constitutes a persistent source of uncertainty that threatens the safe operation of UAVs (Almseidein and Alzidaneen, 2025). Such multi-level heterogeneity leads to inaccurate trajectory prediction and a lack of personalization in air-ground risk quantification, directly compromising UAV operational safety. Developing a systematic computational framework that can perceive the behavioral heterogeneity of ground agents online, quantify air-ground interaction risk in real time, and generate adaptive flight trajectories accordingly has therefore become an urgent need for autonomous UAV operation in heterogeneous multi-agent environments.

In terms of methodologies for low-altitude perception and planning, existing studies have achieved important progress in risk modeling, trajectory prediction, graph-based learning, and trajectory planning. In risk modeling, Zhou et al. (2025) developed a UAV path planning scheme incorporating air-ground third-party risk, embedding UAV crash probability into the third-party risk assessment model. Braßel et al. (2025) achieved substantial reductions in ground risk based on target level of safety constraints. Zheng B. et al. (2025) proposed a collision risk assessment algorithm based on dynamic path prediction and perturbation analysis.

In trajectory prediction, Kim et al. (2025) developed a trajectory prediction and collision risk classification framework for urban air mobility scenarios based on an LSTM-Attention network. Almseidein and Alzidaneen (2025) constructed a three-hidden-layer ANN architecture, providing a highly accurate and efficient solution for real-time UAV trajectory prediction. Tang et al. (2025) integrated pedestrian trajectory prediction with gradient-based planning, improving the safe planning capability of UAVs in crowded dynamic environments.

In addition, graph learning methods provide a new perspective for extracting relational features in complex scenarios, such as low-altitude UAV operations, by explicitly modeling structural relationships among nodes. Ding et al. (2025a) enhanced structural representation through adaptive filtering and graph structure updating, and further improved the stability of graph feature representations by integrating low-pass graph denoising with self-supervised contrastive learning Ding et al. (2025b). These studies indicate that effective modeling of node relationships and graph structures can enhance the representation of complex structural information.

In trajectory planning, Zheng Y. et al. (2025) proposed a two-stage hierarchical 4D low-risk trajectory planning method that effectively addressed multiple risks arising from high-density operations at ultra-low altitude in urban areas. Dong et al. (2025) used ADS-B information to achieve three-dimensional collision-free trajectory planning in low-altitude urban airspace. Gong et al. (2026) integrated deep reinforcement learning with large language models to propose a low-altitude trajectory planning framework that balances safety and economic efficiency.

Nevertheless, the risk models in the above studies mainly focus on factors such as static obstacles, conflicts between aircraft, or ground population exposure, and they generally overlook the dynamic influence of ground agents’ behavioral heterogeneity on air-ground interaction risk. Trajectory prediction studies, in turn, mostly concentrate on the motion of the UAV itself and lack fine-grained perceptual modeling of heterogeneous ground agent behavior. It can thus be seen that existing research has yet to form a closed-loop perception-decision-planning framework for autonomous UAVs operating among heterogeneous ground agents. Such a framework would need to organically integrate three elements: online identification of behavioral styles across multiple types of ground agents, quantification of dynamic air-ground interaction risk, and UAV adaptive trajectory planning. This gap constitutes a major constraint on achieving safe, autonomous UAV operation in heterogeneous multi-agent air-ground environments.

Addressing this problem requires overcoming three interrelated technical challenges: how to identify the behavioral styles of heterogeneous ground agents online; how to fuse behavioral style, predicted trajectory, and air-ground geometric relationships to quantify dynamic air-ground interaction risk; and how to use the resulting perception and risk information to guide UAVs in generating safe, adaptive flight trajectories.

Building on this, this paper proposes a computational intelligence framework for heterogeneity-aware risk perception and risk-adaptive trajectory planning of UAVs operating among heterogeneous ground agents. The framework takes real-time perception of ground agent heterogeneity, quantification of air-ground interaction risk, and adaptive flight trajectory generation as its core objectives. First, a GMM-LightGBM model identifies ground agent driving styles online and drives personalized trajectory prediction. Second, a fuzzy inference system quantifies the spatiotemporal coupling between air and ground agents, generating an interpretable, real-time risk score. Third, an MS-DGST-VNet model predicts UAV flight parameters, and a risk-adaptive cost function dynamically reweights safety, efficiency, and altitude constraints, driving the UAV to generate an adaptive trajectory.

Rather than treating vehicle behavior recognition, risk assessment, and trajectory planning as separate tasks, this study establishes a coupled framework that links ground-vehicle behavioral heterogeneity to UAV planning through personalized trajectory prediction and risk quantification. The main contributions are as follows.

1. A prediction-driven risk perception method considering ground-vehicle behavioral heterogeneity is proposed by integrating driving style recognition with personalized trajectory prediction for air-ground risk quantification.
2. A 3D UAV trajectory planning method integrating maneuver parameter prediction and risk-adaptive decision-making is proposed, where planning weights are dynamically adjusted according to scene risk.
3. Air-ground interaction scenarios are constructed using real-world NGSIM data, and multiple baseline comparisons across different risk scenarios are conducted to validate the key components and overall planning performance.

## 2 Risk perception based on prediction

When UAVs perform missions such as inspection and delivery in urban airspace, their flight safety depends not only on the avoidance of aerial obstacles but also closely on the dynamic state of the ground traffic flow beneath them. Once a UAV malfunctions or loses control and falls, the density, speed, and behavioral style of ground traffic participants will directly determine the severity of the resulting consequences (Chen et al., 2025; Li et al., 2025; Zhang T. et al., 2023).

However, the behavioral heterogeneity of ground traffic participants renders this risk highly dynamic and uncertain. To address this, this section constructs a prediction-driven risk perception module. By identifying the behavioral styles of ground traffic participants and generating personalized trajectories, the module quantifies dynamic air-ground interaction risk. This provides two core types of input to the subsequent planning module, namely, predicted trajectories of ground traffic participants, and a scenario risk score. The module framework is shown in Figure 1. It is noted that our study mainly focuses on the impact of vehicle behavioral heterogeneity on air-ground interaction risk in structured road traffic environments. Therefore, only vehicles are considered as ground traffic participants in the current study.

**FIGURE 1:**
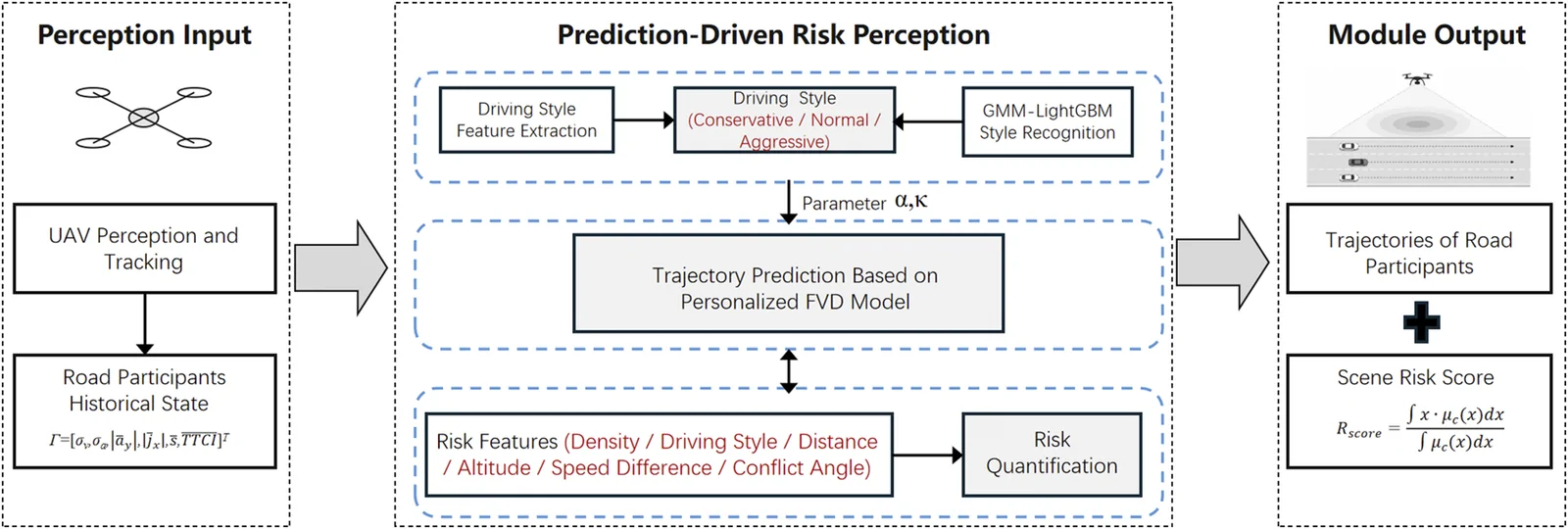
Framework of the risk perception module.

### 2.1 Behavioral style identification

The behavioral style identification of ground traffic participants essentially reflects their long-term driving habits and risk preferences (Wang et al., 2019). Different styles exhibit significant differences in dimensions such as speed control stability, the aggressiveness of acceleration and deceleration, and preferred following distance. These differences directly affect the exposure risk level of ground traffic participants in the event of a UAV crash. To this end, this section constructs a GMM-LightGBM-based online behavioral style identification framework, enabling real-time discrimination of multiple heterogeneous behavior patterns among ground traffic participants within the perception range. The identification process consists of three steps, namely, driving style feature extraction, GMM-based style clustering, and LightGBM-based style identification.

#### 2.1.1 Driving style feature extraction

Driving style is an intrinsic attribute that describes a driver’s driving habits and risk preferences. Building on existing driving behavior modeling approaches (Chen et al., 2024), this study extracts six features from two dimensions, vehicle dynamic characteristics and safety perception, as detailed in Table 1.

**TABLE 1:** Driving style features identification.

Dimension	Feature name	Symbol	Description
Dynamic	Standard deviation of longitudinal velocity	$\sigma_{v}$	Stability of speed control
Dynamic	Standard deviation of longitudinal acceleration	$\sigma_{a}$	Smoothness of acceleration and deceleration
Dynamic	Mean absolute lateral acceleration	$\left|{\bar{a}}_{y}\right|$	Intensity of lane changing or in-lane oscillation
Dynamic	Mean absolute longitudinal jerk	$\left|{\bar{j}}_{x}\right|$	Abruptness of throttle and braking operations
Safety	Mean time headway	$\bar{s}$	Driver’s preferred following distance
Safety	Mean inverse time-to-collision	$\bar{TTCI}$	Potential collision risk with the leading vehicle

The above statistical features are extracted using a short sliding observation window, rather than from a single-frame observation. It is assumed that the perception and tracking module provides the recent historical state sequence of each ground traffic participant, including position, velocity, acceleration, spacing, and relative velocity, which is used to update the six-dimensional driving style feature vector in real time, as shown in Equation 1. For newly detected ground traffic participants, a shorter available window or a default style label is used, until sufficient observations have been accumulated to enable accurate identification.

$$\Gamma ={\left[\sigma_{v},\sigma_{a},\left|{\bar{a}}_{y}\right|,\left|{\bar{j}}_{x}\right|,\bar{s},\bar{TTCI}\right]}^{T}$$ (1)

#### 2.1.2 GMM-based driving style clustering

After quantifying the driving behavior features, this study adopts a Gaussian Mixture Model (GMM) for driving style clustering, in consideration of the complexity of the feature distributions across different styles. GMM models the probability density of n -dimensional feature data as a weighted superposition of K Gaussian components, as shown in Equation 2:

$$p\left(x\right)=\sum_{k=1}^{K}\pi_{k}N\left(\left.x\right|\mu_{k},{Μ}_{k}\right)$$ (2)

where $\pi_{k}$ , $\mu_{k}$ , and ${Μ}_{k}$ denote the mixture weight, mean vector, and covariance matrix of the k -th component, respectively, estimated iteratively through the Expectation-Maximization (EM) algorithm.

Compared with hard clustering algorithms such as K-Means, the soft clustering property of GMM can better capture the multimodal distribution of driving behavior features and the fuzziness of style boundaries. Before clustering, the six-dimensional feature data are standardized using z-score normalization. The optimal number of clusters is then selected from a candidate range $K\in \left\{2,3,...,8\right\}$ according to the Bayesian Information Criterion (BIC), as shown in Equation 3:

$$BIC\left(K\right)=\ln\left(n\right)\cdot d_{k}-2\ln{\hat{L}}_{k}$$ (3)

where n is the sample size, $d_{k}$ is the number of free parameters of the k -th model, and ${\hat{L}}_{k}$ is the corresponding maximum likelihood value.

#### 2.1.3 LightGBM-based style identification

After completing offline driving style labeling using GMM, this study adopts the Light Gradient Boosting Machine (LightGBM) algorithm to achieve online identification of driving style. The model takes the style labels generated by GMM as ground truth, and the LightGBM classifier is trained by minimizing a regularized multi-class cross-entropy loss, as shown in Equation 4:

$$L=-\sum_{i=1}^{N}\sum_{c=1}^{C}y_{ic}\log{\hat{p}}_{ic}+\Omega \left(\theta \right)$$ (4)

where $y_{ic}$ is the truth label indicating whether sample i belongs to class c , ${\hat{p}}_{ic}$ is the predicted class probability of the model, and $\Omega \left(\theta \right)$ is the regularization term.

The model takes the six-dimensional feature vector Γ as input and outputs the driving style category label. Once trained, the classifier can complete style discrimination instantly upon receiving real-time features from the perception system. The identification results simultaneously drive two downstream sub-modules, namely, personalized trajectory prediction and scenario risk quantification.

### 2.2 Trajectory prediction of ground traffic participants

This section constructs a style-driven personalized trajectory prediction module, building on the identification of ground traffic participants’ behavioral styles. When a UAV performs the mission in urban airspace, the area directly beneath it is a multi-lane ground road, with multiple heterogeneous ground vehicles present within the perception range, as shown in Figure 2.

**FIGURE 2:**
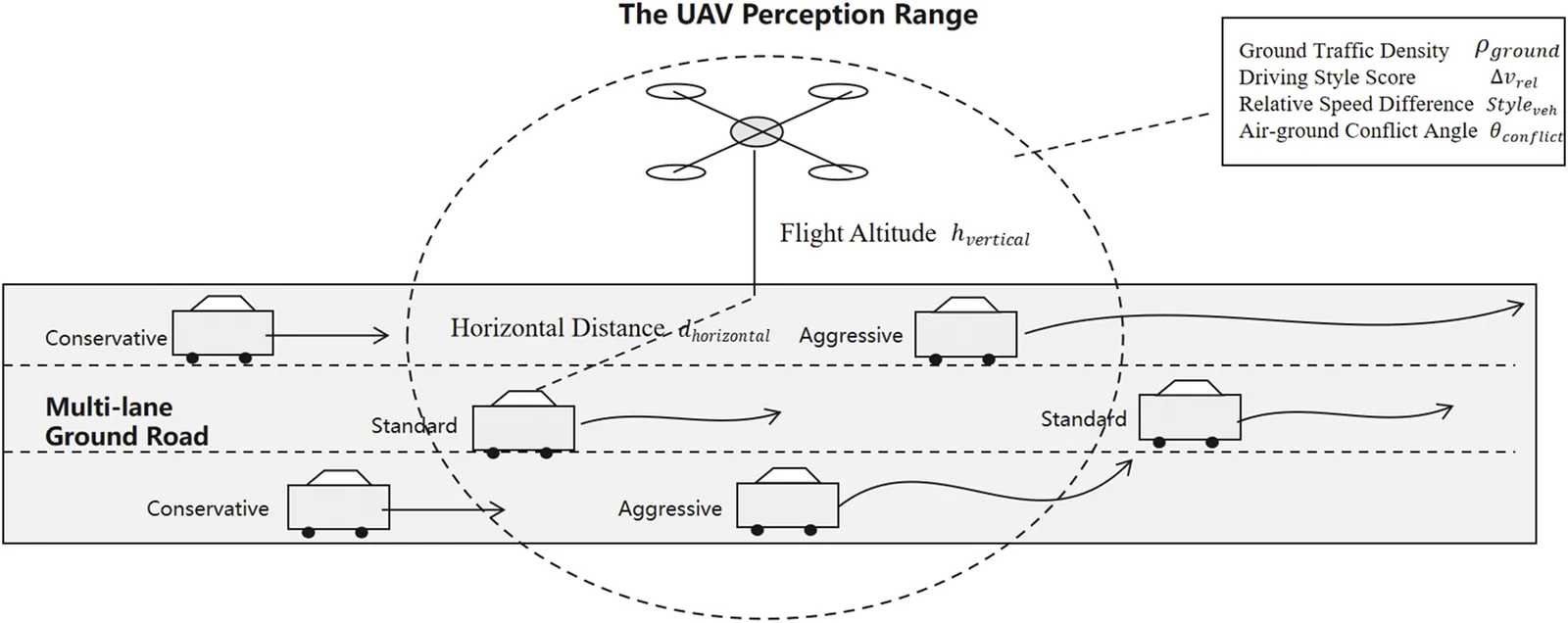
Schematic diagram of the air-ground scenario.

In the air-ground interaction scenario, the longitudinal behaviors of ground traffic participants within the perception range, including acceleration, deceleration, and car-following, are the most critical factors influencing the dynamic variation of ground third party risk. To model these behaviors, this study adopts the Full Velocity Difference (FVD) model (Jiang et al., 2001), which is widely used in traffic flow theory. Its acceleration is calculated as shown in Equation 5:

$$a\left(t\right)=\alpha \left(V_{opt}\left(\Delta x\right)-v\left(t\right)\right)+\kappa \Delta v\left(t\right)$$ (5)

where $v\left(t\right)$ is the speed of the current ground traffic participant (the vehicle closest to the UAV in lateral position), Δx is the spacing from the leading vehicle, and Δv is the relative speed with respect to the leading vehicle. $V_{opt}\left(\Delta x\right)$ is the optimal velocity function for a given spacing, describing the driver’s desired speed, as shown in Equation 6:

$$V_{opt}\left(\Delta x\right)=\frac{V_{\max}}{2}\left[\tan h\left(\frac{\Delta x-s_{0}}{d_{crit}}\right)+1\right]$$ (6)

where $V_{\max}$ , $s_{0}$ , and $d_{crit}$ denote the maximum desired speed, the minimum standstill spacing, and the perception range coefficient, respectively.

The core of this model lies in two personalized parameters. α is the speed recovery coefficient, reflecting how aggressively a driver adjusts toward the optimal speed. κ is the speed-difference response coefficient, characterizing a driver’s sensitivity to changes in relative speed. Participants with different driving styles have different values of parameter combination $\left(\alpha ,\kappa \right)$ . The key to personalized trajectory prediction is to calibrate the optimal parameter combination for each vehicle, which is achieved by minimizing the mean squared error between the FVD-predicted acceleration $a_{pred}$ and the actually observed acceleration $a_{real}$ , which is achieved by minimizing the mean squared error between the FVD-predicted acceleration apred and the actually observed acceleration areal, as shown in Equation 7:

$$\min_{\alpha ,\kappa }MSE\left(\alpha ,\kappa \right)=\frac{1}{N}\sum_{i=1}^{N}{\left(a_{pred,i}\left(\alpha ,\kappa \right)-a_{real,i}\right)}^{2}$$ (7)

Since the relationship between the objective function and the parameters $\left(\alpha ,\kappa \right)$ is complex and may involve multiple local optima, this study employs a Genetic Algorithm (GA) for global parameter optimization. The optimization is run separately for each driving style, yielding a set of personalized FVD model parameters for each.

During trajectory prediction, the UAV first identifies the driving style of each ground traffic participant within the perception range online, and then invokes the corresponding personalized FVD model. Iterative forward integration is performed with a time step of 0.1 s, generating the predicted longitudinal velocity $v_{pred}$ and longitudinal position $x_{pred}$ for the next few seconds. For the lateral position $y_{pred}$ , a simplified assumption is adopted. Each participant maintains its current lateral position unchanged throughout the prediction horizon. This assumption is applicable to the lane-constrained scenarios considered in this study. For non-lane-constrained environments, the current prediction method needs to be further extended to 2D/3D trajectory prediction.

### 2.3 Risk quantification of air-ground interaction

After obtaining the behavioral styles and personalized predicted trajectories of ground traffic participants, this section constructs a risk quantification model for air-ground interaction scenarios. The air-ground interaction risk referred to in this study denotes the potential third party ground safety risk arising during UAV operation from the state of ground traffic flow within the perception range, including vehicle density, driving style, and dynamic behavior. This risk is specifically reflected as the expected loss inflicted on ground traffic participants by the UAV under abnormal conditions (Yáng et al., 2025; Melnyk et al., 2014). Unlike traditional low-altitude planning approaches that model risk only in terms of static obstacles or aerial collisions, this study incorporates the behavioral heterogeneity of ground traffic participants into the risk quantification system, enabling the risk assessment to respond dynamically to real-time changes in ground traffic conditions.

To fully characterize air-ground interaction risk, this study constructs a six-dimensional air-ground risk feature vector $V_{risk}^{ag}$ from three perspectives, the macroscopic traffic environment, the behavioral characteristics, and the air-ground geometric relationship, as shown in Equation 8.

$$V_{risk}^{ag}=\left[\rho_{ground},{Style}_{veh},d_{horizontal},h_{vertical},{\Delta v}_{rel},\theta_{conflict}\right]$$ (8)

where $\rho_{ground}$ is the ground traffic density within the UAV’s perception range. ${Style}_{veh}$ is the driving style score of the nearest ground traffic participant, with the aggressive style assigned the highest value. $d_{horizontal}$ is the horizontal distance between the UAV and the nearest ground traffic participant. $h_{vertical}$ is the current flight altitude of the UAV, where a lower flight altitude corresponds to a higher exposure risk for ground traffic participants. ${\Delta v}_{rel}$ is the average relative speed difference between the UAV and the ground traffic participant. $\theta_{conflict}$ is the angle between the UAV’s heading and the motion direction of the nearest ground traffic participant, reflecting the geometric configuration of the air-ground conflict.

To further emulate the fuzzy perception and qualitative judgment process of a UAV operator regarding the flight environment, this study constructs a risk quantification model based on a Fuzzy Logic System (FLS). The model sequentially performs three steps.

1. Fuzzification. Membership functions are used to convert the precise input values of the six-dimensional risk features into membership degrees within fuzzy sets. Taking the horizontal distance $d_{horizontal}$ as an example, three fuzzy sets are defined {near, medium, far} using triangular membership functions. The flight altitude $h_{vertical}$ is defined with three fuzzy sets {low, medium, high}. The driving style variable is mapped directly to its corresponding style-label fuzzy set, and the remaining continuous variables are likewise fuzzified using their respective membership functions.
2. Rule base. A fuzzy rule base composed of IF-THEN rules is constructed, following six categories of safety principles: ①the proximity principle, where risk increases as horizontal distance decreases. ②the behavioral aggressiveness principle, where risk increases with more aggressive driving styles. ③the speed synchronization principle, where risk increases with a larger relative speed difference. ④the density principle, where risk increases with higher ground traffic density. ⑤the vertical separation principle, where risk increases as flight altitude decreases. ⑥the three-dimensional conflict geometry principle, where risk increases as the air-ground conflict angle approaches the vertical direction. A representative rule is given below. If $\rho_{ground}$ is high, ${Style}_{veh}$ is aggressive, $d_{horizontal}$ is near, $h_{vertical}$ is low, ${\Delta v}_{rel}$ is large, and $\theta_{conflict}$ is close to the vertical angle, then the risk level is extremely high.

It should be noted that the current fuzzy rules are manually constructed based on the above safety principles and the physical meanings of the risk factors, providing good interpretability. However, the specification of the rules and membership functions still relies to some extent on prior knowledge, and their adaptability to more complex traffic scenarios can be further improved.

3. Fuzzy inference and defuzzification. Fuzzy inference and defuzzification are performed using the classical Mamdani inference method. For each rule, the AND operator, taken as the minimum function, is used to compute the activation strength of the antecedent. This strength is then used to truncate the membership function of the rule’s consequent, producing an individual fuzzy output $\mu_{i}^{\prime }\left(x\right)$ for each rule. The fuzzy outputs of all rules are then aggregated using the maximum method, as shown in Equation 9.

$$\mu_{c}\left(x\right)=\overset{N}{\underset{i=1}{⋃}}\mu_{i}^{\prime }\left(x\right)=\max\left(\mu_{i}^{\prime }\left(x\right)\right)$$ (9)

The aggregated fuzzy output is then defuzzified using the center of area (COA) method, yielding a precise risk score $R_{score}\in \left[0,1\right]$ , where a higher value indicates greater risk, as shown in Equation 10:

$$R_{score}=\frac{\int x\cdot \mu_{c}\left(x\right)dx}{\int \mu_{c}\left(x\right)dx}$$ (10)

In summary, the prediction-driven risk perception module constructed in this section converts dynamic ground environment information into two core outputs for the subsequent planning module. The first output is the personalized predicted trajectory of each ground traffic participant within the perception range, which is used to constrain the UAV’s sampling range and reduce collision probability. The second output is the quantified scenario risk score $R_{score}$ , which provides an interpretable macroscopic assessment of the current scenario’s risk level, enabling the planning module to achieve genuinely scenario-adaptive decision making.

## 3 Adaptive trajectory planning

Based on the two types of outputs obtained from the risk perception module, this section constructs a low-altitude adaptive trajectory planning framework driven by dynamic risk perception. Traditional low-altitude trajectory planning methods mainly focus on static obstacles or conflicts between aircraft when modeling risk, overlooking the influence of the dynamic behavior of ground traffic flow on flight safety (Gutierrez-Martinez et al., 2025; Zhang and Xu, 2025). These methods also lack a closed-loop mechanism that organically integrates behavioral perception information with planning decisions. To address this, this section proposes a planning framework comprising three stages. The first stage is the construction of a low-altitude dynamic risk field based on ground risk perception. The second stage is maneuver parameter prediction based on MS-DGST-VNet. The third stage is risk-adaptive microscopic trajectory optimization.

### 3.1 Low-altitude dynamic risk field construction

The ground space within the UAV’s perception range is discretized into an M×N grid map, as shown in Figure 3. Each grid cell $\left(i,j\right)$ corresponds to a Δl×Δl region on the ground. For each grid cell, its center point is taken as a virtual UAV position, and the fuzzy logic risk quantification system is invoked to compute the corresponding risk value. This is then fused with forward-looking information from the predicted trajectories, as shown in Equation 11.

$$R\left(i,j,t\right)=\left(1-\lambda \right)\cdot R_{score}\left(i,j,t\right)+\lambda \cdot \frac{1}{T_{h}}\sum_{\tau =1}^{T_{h}}R_{score}\left(i,j,t+\tau \right)$$ (11)

where $R\left(i,j,t\right)$ is the integrated risk score of grid cell $\left(i,j\right)$ at time t . $R_{score}\left(i,j,t+\tau \right)$ is the expected risk score at time t+τ , estimated from the predicted trajectory generated by the FVD model. $T_{h}$ is the prediction horizon, and $\lambda \in \left[0,1\right]$ is the lookahead weighting coefficient. This formula combines the current risk with the future expected risk through weighted fusion, giving the risk field a forward-looking temporal depth and providing the UAV with a dynamic spatial risk reference.

**FIGURE 3:**
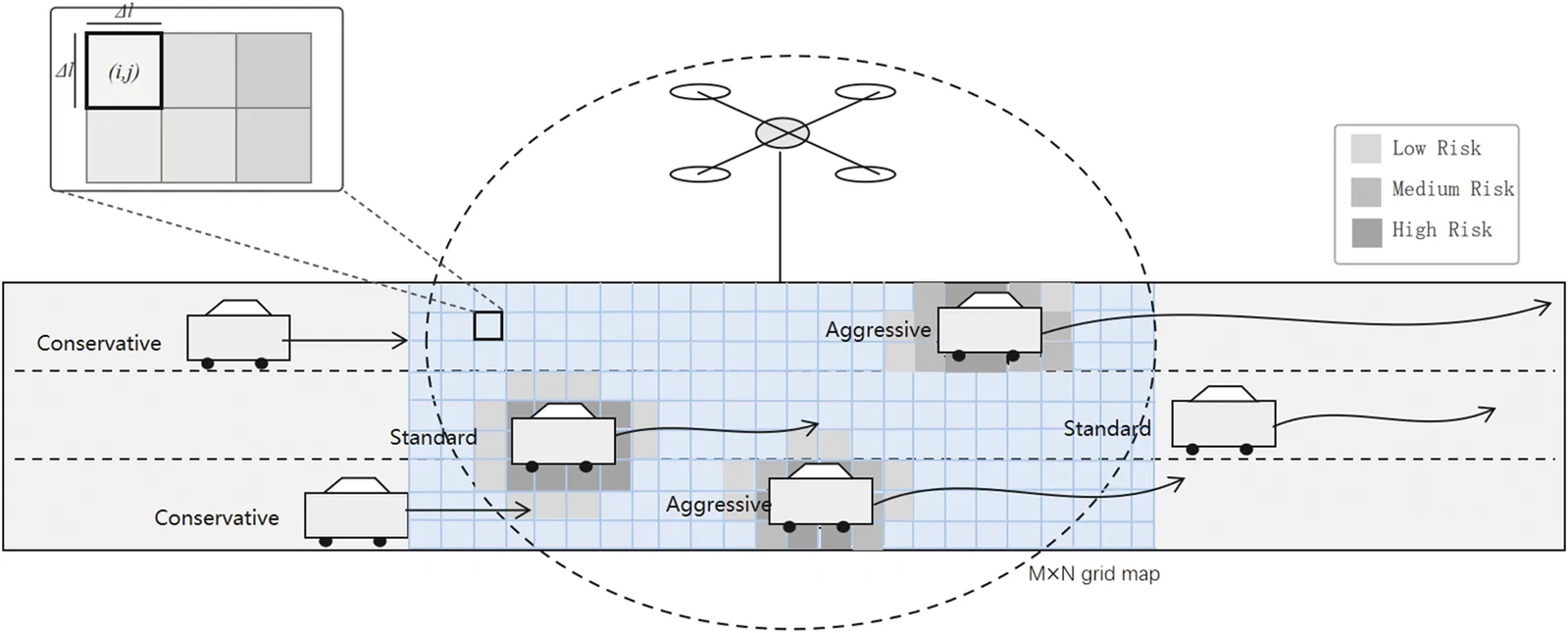
Risk field construction.

After this, the predetermined sequence of waypoints is evaluated for risk based on the dynamic risk field. For the m -th waypoint $p_{m}=\left(x_{m},y_{m},z_{m}\right)$ , its ground-projected risk value is defined as shown in Equation 12:

$$R_{wp}\left(m\right)=R\left(i_{m},j_{m},t_{m}\right)$$ (12)

where $\left(i_{m},j_{m}\right)$ are the grid coordinates of the waypoint’s ground projection, and $t_{m}$ is the time at which the UAV is expected to reach this waypoint. When $R_{wp}\left(m\right)$ exceeds a preset risk threshold $R_{th}$ , an altitude-raising strategy is triggered, as shown in Equation 13:

$${z_{m}}^{\prime }=z_{m}+\Delta h_{\max}\cdot \frac{R_{wp}\left(m\right)-R_{th}}{1-R_{th}},{z_{m}}^{\prime }\leq h_{\max}$$ (13)

where ${z_{m}}^{\prime }$ is the adjusted altitude, and $\Delta h_{\max}$ is the maximum altitude adjustment. This mechanism enables the UAV to automatically climb to a safer altitude when flying over high-risk ground areas, while maintaining cruising altitude in low-risk areas to ensure mission efficiency, thereby achieving risk-adaptive route adjustment at the macroscopic level.

### 3.2 Maneuver parameter prediction based on MS-DGST-VNet

After completing the risk field construction and waypoint altitude adjustment, this section constructs a trajectory parameter prediction model to predict the key parameters of the UAV’s flight trajectory. These parameters are the planned duration $\Delta T_{pred}$ and the three-dimensional displacement $\left[\Delta X_{pred},\Delta Y_{pred},\Delta Z_{pred}\right]$ . The model input is defined as the UAV’s motion features and the road network environment features. The UAV’s motion features consist of the historical trajectory sequence from the several seconds preceding the maneuver decision, where the feature vector $h_{f}$ at each time step is composed of position $\left(x,y,z\right)$ , velocity $\left(v_{x},v_{y},v_{z}\right)$ , and acceleration $\left(a_{x},a_{y},a_{z}\right)$ , capturing the dynamic evolution pattern of the UAV. The road network environment features consist of a sequence of geometric information along the flight path, where the feature vector $h_{e}$ at each spatial node is composed of coordinates $\left(x,y,z\right)$ , curvature κ , and route segment ID, providing the model with spatial constraint information. In addition, the sequence of risk scores $R_{score}$ along the route is incorporated as an additional input feature, enabling the model to perceive the influence of ground traffic conditions on maneuver parameters.

For this trajectory parameter prediction task, this study proposes the MS-DGST-VNet model (Maneuver-Specific Dual-Graph Spatio-Temporal VectorNet). The model architecture is shown in Figure 4 and consists of the following three core stages.

1. Polyline subgraph network

**FIGURE 4:**
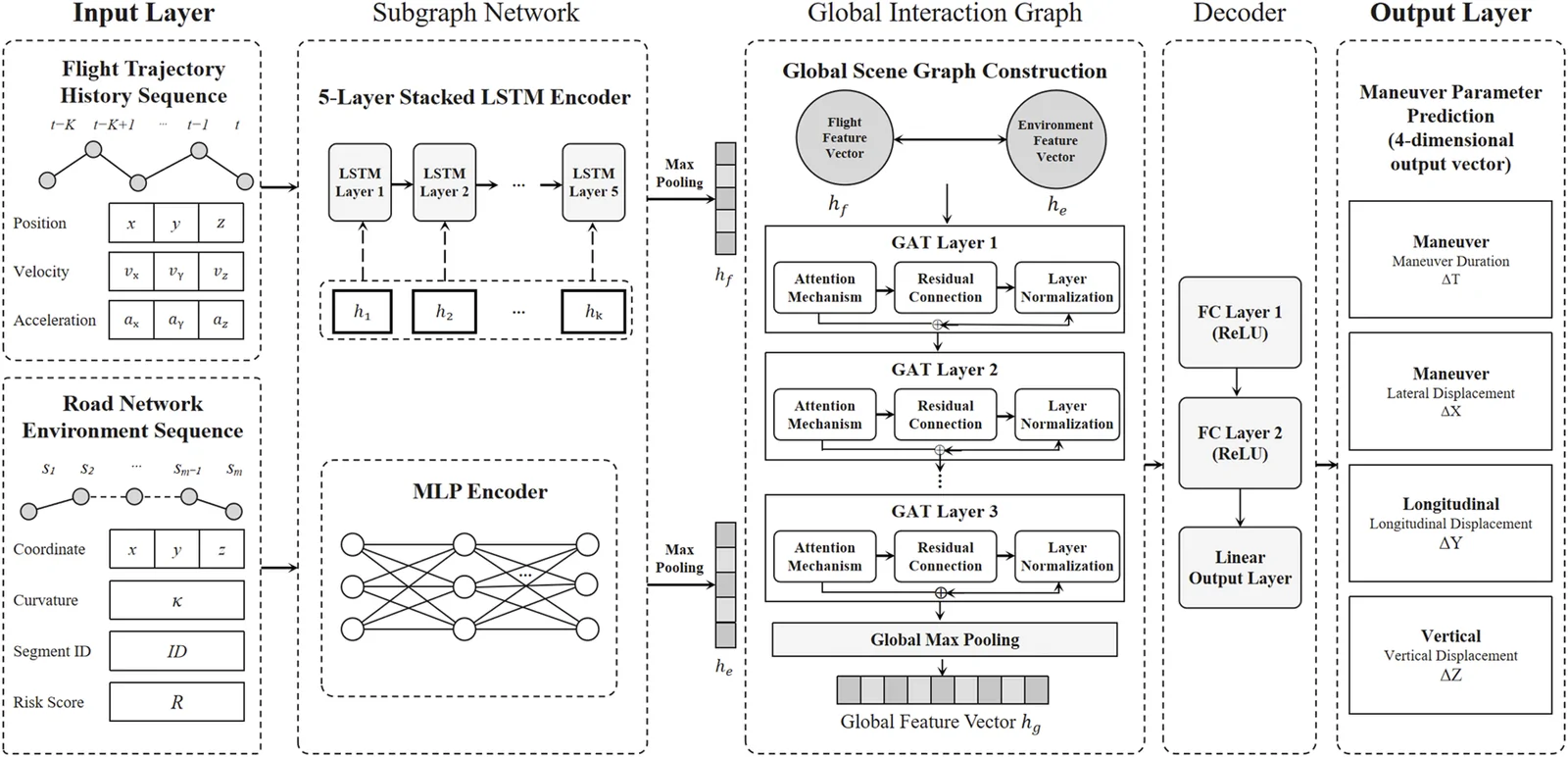
Architecture of the MS-DGST-VNet model.

The polyline subgraph network independently encodes the two heterogeneous sequence inputs. For the UAV’s historical trajectory sequence, given its strong long-range temporal dependency, a five-layer stacked LSTM network is used for encoding. This effectively captures the dynamic evolution pattern within the trajectory sequence and replaces the MLP-based aggregation used in the original VectorNet. For the road network geometric data, a multilayer perceptron (MLP) is used to extract node sequence features. Through max pooling, the UAV trajectory and the route geometry are each encoded into fixed-dimensional feature vectors $P_{agent}$ and $P_{lane}$ .

2. Global interaction graph network

Graph-based relational modeling can enhance structural feature representation by explicitly capturing the connectivity relationships among nodes (Liang et al., 2026). Based on this, $P_{agent}$ and $P_{lane}$ are treated as nodes of a higher-level global scene graph, and a three-layer stacked Graph Attention Network (GAT) is constructed for interaction modeling. By stacking multiple graph attention modules and incorporating residual connections and layer normalization, the model can capture deep, high-order interaction patterns between the UAV and its environment. The output features of all nodes are aggregated through max pooling into a global feature vector $h_{g}$ , which is then passed to the decoder.

3. Decoder and output layer

The decoder consists of two fully connected layers, mapping $h_{g}$ to a 256-dimensional hidden feature $h_{decode}$ . The output layer performs a linear transformation, mapping $h_{decode}$ to a four-dimensional output vector $\left[\Delta T_{pred},\Delta X_{pred},\Delta Y_{pred},\Delta Z_{pred}\right]$ . The total loss of the model is a weighted sum of the MSE losses for each prediction target, as shown in Equation 14:

$$L_{total}=\alpha \cdot {MSE}_{T}+\beta \cdot {MSE}_{\Delta X}+\gamma \cdot {MSE}_{\Delta Y}+\delta \cdot {MSE}_{\Delta Z}$$ (14)

where α=β=γ=δ=1 are hyperparameters that balance the subtasks and can be adjusted according to task requirements.

### 3.3 Risk-adaptive trajectory planning

Guided by the waypoints and the predicted maneuver parameters, this section constructs a risk-adaptive microscopic trajectory optimization module to generate smooth, safe, and dynamically feasible flight trajectory between adjacent waypoints. This module injects the scenario risk score $R_{score}$ into three key stages, trajectory sampling, cost evaluation, and safety verification, achieving genuinely risk-adaptive planning.

#### 3.3.1 Three-dimensional trajectory sampling

The predicted maneuver parameters $[\Delta T_{pred},\Delta X_{pred},\Delta Y_{pred},$ $\Delta Z_{pred}]$ are used to constrain the sampling range of candidate trajectories, transforming the traditional high-dimensional unconstrained search into a prediction-guided, low-dimensional directional search.

The temporal sampling set T : The terminal time is sampled from an interval centered on the predicted maneuver duration $\Delta T_{pred}$ , with the sampling range dynamically adjusted according to the risk score.

$$T=\left\{T_{i}|T_{i}\in \left[\Delta T_{pred}\left(1-\delta_{T}\right),\Delta T_{pred}\left(1+\delta_{T}\right)\right],i=1,...,N_{T}\right\}$$ (15)

where $\delta_{T}$ is the temporal perturbation factor. A smaller value is used under high risk to narrow the search range, while a larger value is used under low risk to increase diversity.

The lateral sampling set D : The lateral offset is sampled around $\Delta Y_{pred}$ , with the offset range dynamically constrained by the predicted trajectories of surrounding participants.

$$d_{k}=\Delta Y_{pred}+\left(-d_{\max}+\frac{2d_{\max}}{N_{d}-1}\cdot k\right),k=0,1,...,N_{d}-1$$ (16)

$$d_{\max}=\max\left(0.5,d_{safe}-\Delta d_{pred}\right)$$ (17)

where $d_{safe}$ is the lateral safety margin, and $\Delta d_{pred}$ is the predicted lateral offset of surrounding participants.

The vertical sampling set Z : The vertical offset is sampled around $\Delta Z_{pred}$ , with sampling biased toward the positive direction under high risk to encourage climbing.

$$Z=\left\{z_{k}|z_{k}\in \left[z_{ref}+\Delta Z_{pred}-\delta_{z}^{-},z_{ref}+\Delta Z_{pred}+\delta_{z}^{+}\right]\right\}$$ (18)

Under high risk $\delta_{z}^{+}>\delta_{z}^{-}$ , biasing the sampling toward the climbing direction. Under low risk $\delta_{z}^{+}=\delta_{z}^{-}$ , giving symmetric sampling to ensure diversity.

#### 3.3.2 Risk-adaptive cost function

A risk-adaptive cost function is constructed to comprehensively evaluate each candidate trajectory, as shown in Equation 19.

$$J_{total}^{UAV}=w_{task}J_{task}+w_{safe}\left(R_{score}\right)J_{safe}+w_{alt}\left(R_{score}\right)J_{altitude}+w_{eff}J_{eff}+w_{smooth}J_{smooth}$$ (19)

where the task cost $J_{task}$ is the squared distance error between the trajectory’s terminal position $P_{end}$ and the target waypoint $P_{target}$ . The safety cost $J_{safe}$ is a penalty based on the minimum distance between the trajectory $P_{UAV}$ and the predicted trajectories ${\hat{P}}_{k}$ of ground traffic participants. The altitude constraint cost $J_{altitude}$ is a penalty for the trajectory’s deviation from the cruising altitude $h_{cruise}$ . The efficiency cost $J_{eff}$ is the maneuver completion time ΔT . The smoothness cost $J_{smooth}$ is the trajectory’s jerk. The calculation formulas for each cost term are given below.

$$J_{task}\left.=\right|P_{end}-P_{target}{|}_{2}$$ (20)

$$J_{safe}=\sum_{k}\max{\left(0,d_{safe}-\min_{t}\left|P_{UAV}\left(t\right)-{\hat{P}}_{k}\left(t\right)\right|\right)}^{2}$$ (21)

$$J_{altitude}=\frac{1}{N}\sum_{i=1}^{N}{\left(z_{i}-h_{cruise}\right)}^{2}$$ (22)

$$J_{eff}=\Delta T$$ (23)

$$J_{smooth}=\frac{1}{N}\left.\sum_{i=1}^{N}\right|{\dot{a}}_{i}{|}^{2}$$ (24)

Among the weight coefficients, $w_{safe},w_{alt},w_{eff}$ are dynamically adjusted according to the scenario risk score $R_{score}$ , as shown in Equations 25–27.

$$w_{safe}\left(R_{score}\right)=W_{safe,base}+\frac{W_{safe,\max}-W_{safe,base}}{1+e^{-k\left(R_{score}-R_{mid}\right)}}$$ (25)

$$w_{eff}\left(R_{score}\right)=W_{eff,base}\left(1-\frac{w_{safe}\left(R_{score}\right)-W_{safe,base}}{W_{safe,\max}-W_{safe,base}}\right)$$ (26)

$$w_{alt}\left(R_{score}\right)=W_{alt,base}\left(1+\frac{w_{safe}\left(R_{score}\right)-W_{safe,base}}{W_{safe,\max}-W_{safe,base}}\cdot 0.5\right)$$ (27)

Among the candidate trajectories that pass the safety verification, the one with the minimum total cost $J_{total}^{UAV}$ is selected as the optimal trajectory and executed by the UAV’s flight control system.

$$\forall t\in \left[0,T_{sample}\right],\left|P_{UAV}\left(t\right)-{\hat{P}}_{k}\left(t\right)\right|\geq d_{safe}$$ (28)

To clearly illustrate the data flow among different modules and the overall execution process, the proposed heterogeneity-aware risk-adaptive UAV trajectory planning method is summarized in Algorithm 1.

Algorithm 1 “Heterogeneity-Aware Risk-Adaptive UAV Trajectory Planning”

**Input:** Vehicle behavioral features Γ ; UAV motion feature $h_{f}$ ; Road-network feature $h_{e}$

**Output:** Optimal UAV trajectory ${P*}_{UAV}$ ​
1: Initialization;
2: **for** *i* = 1, 2, …, N do
3:  Style_*i* ← LightGBM( Γ_i );
4: $\left(\alpha \_i,\kappa \_i\right)$ ← Personalized FVD (Style_*i*)_;
5: ${Trj}_{veh\_i}$ ← FVD $\left(\alpha \_i,\kappa \_i\right)$ ← Vehicle trajectory prediction
6: **end for**
7: $R_{score}$ ← $V_{risk}^{ag}=\left[\rho_{ground},{Style}_{veh},d_{horizontal},h_{vertical},{\Delta v}_{rel},\theta_{conflict}\right]$ ;
8: $R\left(i,j,t\right)$ ← $DynamicRiskField\left(R_{score},{Trj}_{veh\_i}\right)$ ;
9: $R_{wp}\left(m\right)=R\left(i_{m},j_{m},t_{m}\right)$ ;
10: **if** $R_{wp}\left(m\right)$ > $R_{th}$ then
11: ${z_{m}}^{\prime }$ ← $z_{m}+\Delta h_{\max}\cdot \frac{R_{wp}\left(m\right)-R_{th}}{1-R_{th}}$ ;
12: **end if**
13: [ $\Delta T_{pred}$ ; $\Delta X_{pred},\Delta Y_{pred},\Delta Z_{pred}$ ]← MS-DGST-VNet ( $h_{f}$ , $h_{e}$ , $R_{score}$ , ${z_{m}}^{\prime }$ );
14: Generate candidate trajectories $P_{UAV}$ according to Equations 15–18;
15: Update $w_{safe}\left(R_{score}\right)$ , $w_{eff}\left(R_{score}\right)$ , $w_{alt}\left(R_{score}\right)$ ;
16: **for** each $P_{UAV}$ do
17: $J_{total}^{UAV}$ ← Equations 19–27
18:  Verify $\left|P_{UAV}\left(t\right)-{\hat{P}}_{k}\left(t\right)\right|\geq d_{safe}$ ← Equation 28
19: **end for**
20: ${P*}_{UAV}$ ← arg min $J_{total}^{UAV}$ ;
21: **return** ${P*}_{UAV}$ .

In summary, the adaptive trajectory planning framework constructed in this chapter, based on dynamic risk perception, uses the ground dynamic risk field as the route reference, the MS-DGST-VNet maneuver parameter prediction as the planning guidance, and the risk-adaptive cost function as the core evaluation mechanism. This achieves a complete closed loop from ground heterogeneity perception to low-altitude safety planning, providing systematic planning methodological support for the safe and adaptive operation of autonomous UAVs in air-ground intelligent transportation systems.

## 4 Experimental validation and analysis

### 4.1 Experimental setup

The experiments in this study use a air-ground intelligent transportation system scenario as the core validation environment. The scenario is set as follows: A UAV performing a highway infrastructure inspection mission flies along a predetermined route at an altitude of 50–120 m. It is required to perceive the behavioral styles of participants in the mixed ground traffic flow, assess the air-ground interaction risk, and adaptively adjust its flight altitude and speed when the risk is high.

The simulation platform comprises two levels, ground traffic flow simulation and UAV flight simulation. The ground traffic flow simulation data are extracted from the real-world NGSIM dataset (US-101 highway segment). After filtering and preprocessing, complete trajectory records for 51 vehicles are obtained, with a time step of 0.1 s, yielding 12,195 time-series vehicle state records. The road scenario is a three-lane highway with a lane width of 3.75 m. Based on the constructed ground simulation segments, 130 UAV air-ground interaction risk assessment scenarios are further constructed. The UAV’s motion parameters are set with reference to the operating characteristics of typical civilian multi-rotor UAVs, with a flight altitude range of 50–120 m, an average cruising speed of 14 m/s, and a perception radius of 150 m.

The simulation platform is built on Python 3.10 and runs on a workstation equipped with an Intel Core i9-13900K processor, 32 GB of DDR5 memory, and an NVIDIA GeForce RTX 4090 GPU, with Ubuntu 22.04 LTS as the operating system. The main dependencies include NumPy 1.24, Pandas 2.0, Scikit-learn 1.3, and SciPy 1.11. The MS-DGST-VNet model is trained using the PyTorch 2.1 framework, with the Adam optimizer used during training. The key experimental parameter settings are shown in Table 2.

**TABLE 2:** Parameter settings.

Module	Parameter	Symbol	Value
Clustering	Candidate cluster number range	K	{2, 3, … , 8}
	Optimal cluster number (BIC)	K *	3
FVD model	Maximum desired speed	$V_{\max}$	30.0 m/s
	Minimum standstill spacing	$s_{0}$	2.0 m
	Perception range coefficient	$d_{crit}$	15.0 m
	Prediction time step	Δt	0.1 s
Dynamic risk field	Lookahead weighting coefficient	λ	0.4
	Prediction horizon	$T_{h}$	3.0 s
	Grid cell size	Δl	2 m
	Maximum altitude adjustment	$\Delta h_{\max}$	30 m
	Risk trigger threshold	$R_{th}$	0.3
MS-DGST-VNet	Initial learning rate	—	1 × 10^−3^
	Batch size	—	32
	Maximum training epochs	—	200
Trajectory sampling	Number of temporal samples	$N_{T}$	5
	Number of lateral samples	$N_{d}$	5
	Lateral safety margin	$d_{safe}$	3.5 m

* denotes the optimal number of clusters determined according to the Bayesian Information Criterion (BIC).

### 4.2 Perception module validation

#### 4.2.1 Behavioral style identification

From the complete trajectory records of 51 vehicles extracted from the NGSIM dataset, a sliding observation window is applied to the time-series state sequence of each vehicle to extract the six-dimensional driving style feature vector. The window length is set to 3 s (30 time steps), with a sliding step of 1.5 s. Within each window, the six-dimensional statistical features ${\left[\sigma_{v},\sigma_{a},\left|{\bar{a}}_{y}\right|,\left|{\bar{j}}_{x}\right|,\bar{s},\bar{TTCI}\right]}^{T}$ are computed. The average trajectory duration per vehicle is approximately 23.9 s, yielding about 14 windows per vehicle. In total, 735 feature samples are extracted from the 51 vehicles.

Based on the Bayesian Information Criterion (BIC), the optimal value is selected from the candidate cluster number range K∈{2,3,…,8}, resulting in K = 3 as the optimal number of clusters. This corresponds to three driving style categories, conservative, standard, and aggressive, which align well with the risk perception requirements of low-altitude scenarios. The clustering results yield 252 conservative samples (34.3%), 372 standard samples (50.6%), and 111 aggressive samples (15.1%). Figure 5 visualizes the first two principal components obtained from principal component analysis (PCA) of the six-dimensional driving style features. PC1 and PC2 together explain 82.9% of the cumulative variance, and the three styles exhibit clear linear separation in the principal component space, validating the effectiveness of the GMM clustering results.

**FIGURE 5:**
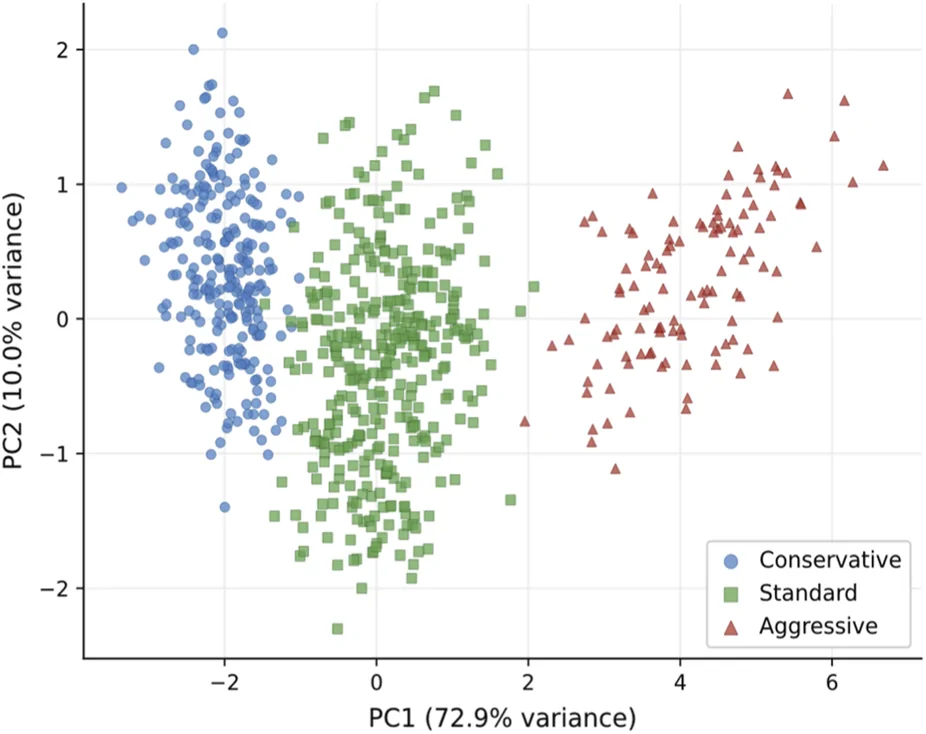
Driving style clustering results.

The three styles exhibit clear separation boundaries in the feature space, with $\sigma_{a}$ and $\bar{TTCI}$ being the most discriminative features. In this section, the Kruskal–Wallis test is used to examine the overall differences among the three styles, and pairwise comparisons are conducted using the Mann-Whitney U test, with the effect size $\left|r\right|$ used to measure the practical significance of the differences. As shown in Figure 6, the between-group differences in $\sigma_{a}$ (H = 498.7) and $\bar{TTCI}$ (H = 480.6) are both significant at the 0.001 level (p < 0.001), with effect sizes $\left|r\right|$ exceeding 0.79. This indicates that the three styles are highly distinguishable, consistent with the physical understanding of driving behavior.

**FIGURE 6:**
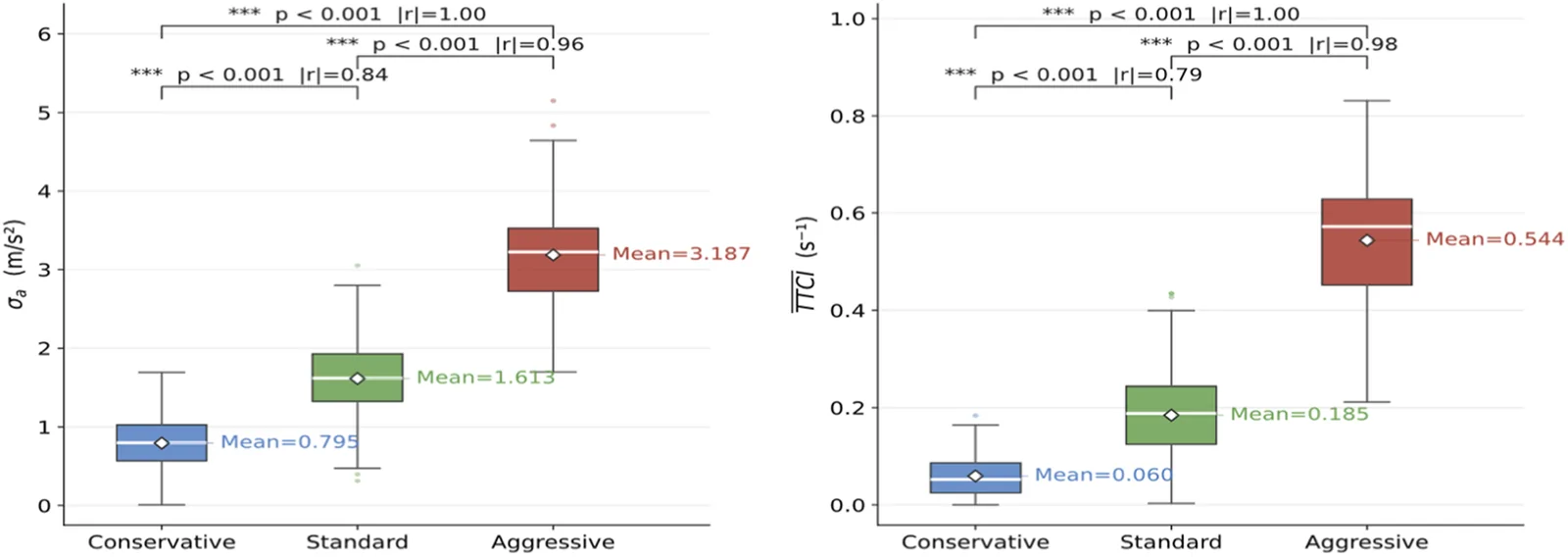
Boxplots of driving style results.

Using the GMM cluster labels as ground truth, the data are split into training and test sets at a 70/30 ratio. After addressing class imbalance in the training set through resampling, a LightGBM gradient boosting classifier is trained, with the identification performance shown in Table 3. The classifier achieves an overall accuracy of 0.96 and a Macro-F1 score of 0.96, with the F1 scores of all three styles exceeding 0.91. This validates the effectiveness of the GMM-LightGBM two-stage framework for the behavioral style identification of ground traffic participants. The conservative style is identified most accurately (Recall = 1.00, F1 = 0.99), followed by the standard style (F1 = 0.97), while the aggressive style achieves an F1 of 0.91. This indicates that the model retains strong discriminative ability for high-risk styles, helping the UAV identify high-risk ground traffic participants in a timely manner. The confusion matrix (Figure 7) further shows that four aggressive samples are misclassified as standard, and three standard samples are misclassified as other categories. In total, seven samples are misclassified, corresponding to an overall misclassification rate of only 3.2%.

**TABLE 3:** Behavioral style identification performance.

Driving style	Precision	Recall	F1-score	Support
Conservative	0.99	1.00	0.99	76
Standard	0.96	0.97	0.97	112
Aggressive	0.94	0.88	0.91	33
Macro average	0.96	0.95	0.96	221

**FIGURE 7:**
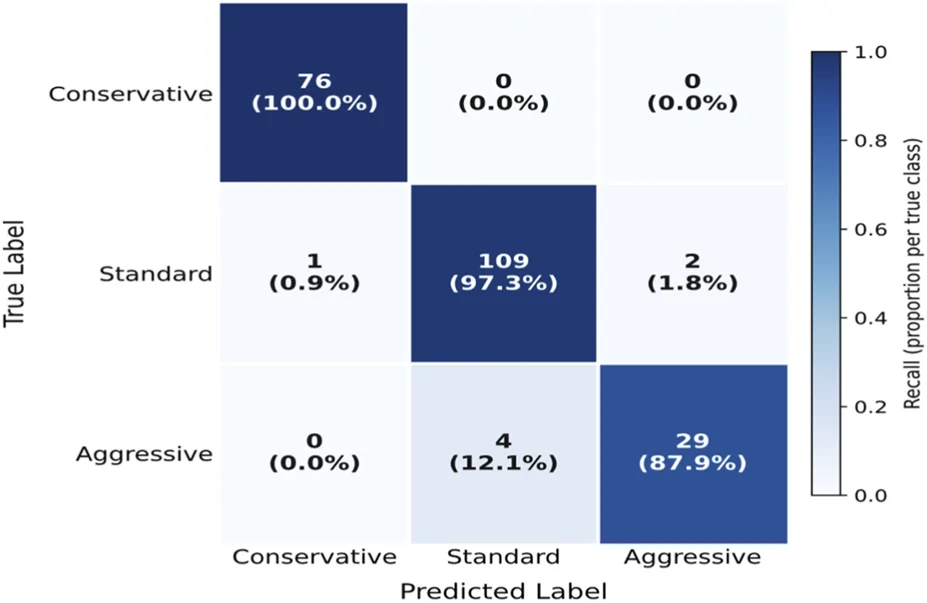
Confusion matrix of behavioral style identification.

#### 4.2.2 Personalized trajectory prediction validation

Based on the identified driving styles, the Genetic Algorithm (GA) is used to individually calibrate the FVD model parameters $\left(\alpha ,\kappa \right)$ for each driving style. The calibration results and prediction accuracy are shown in Table 4. Both α and κ exhibit a monotonically positive correlation with the aggressiveness of the driving style. The speed recovery coefficient α increases from 0.31 for the conservative style to 0.53 for the aggressive style, with the corresponding speed response time constant 1/ α decreasing from 3.23 s to 1.89 s, reflecting the faster speed adjustment response of aggressive drivers. κ increases from 0.18 to 0.33, indicating the higher sensitivity of aggressive drivers to changes in relative speed. These parameter differences indicate distinct dynamic response characteristics among different driving styles during vehicle-following behavior, providing a basis for style-specific personalized motion prediction.

**TABLE 4:** Comparison of trajectory prediction performance.

Driving style	Method	α	κ	1/ α	MSEa (m/s^2^)	RMSEv (m/s)	RMSEx (m)
Conservative	Personalized FVD (ours)	0.310	0.180	3.23	0.0124	0.0186	1.10
	Unified FVD	0.394	0.274	2.54	0.0137	0.0198	1.18
Standard	Personalized FVD (ours)	0.380	0.220	2.63	0.0213	0.0221	1.37
	Unified FVD	0.394	0.274	2.54	0.0215	0.0217	1.36
Aggressive	Personalized FVD (ours)	0.530	0.330	1.89	0.0387	0.0253	1.49
	Unified FVD	0.394	0.274	2.54	0.0446	0.0278	1.68

To further quantify the contribution of driving-style personalization to trajectory prediction performance, Unified FVD is introduced as a non-personalized baseline. Unlike Personalized FVD, which calibrates separate parameters for conservative, standard, and aggressive driving styles, Unified FVD combines the training samples from all three driving styles and uses the same Genetic Algorithm (GA) for parameter calibration. Specifically, the mean squared error (MSE) between the FVD-predicted acceleration and the actual acceleration is minimized under the same parameter search ranges and GA settings, resulting in unified parameters of α = 0.394 and κ = 0.274. To ensure a fair comparison, the two models use the same FVD structure, parameter calibration method, and trajectory prediction settings, with the only difference being whether style-specific parameters are employed. The acceleration mean squared error (MSEa), velocity root mean squared error (RMSEv), and longitudinal position root mean squared error (RMSEx) are used to evaluate the prediction performance. The comparison results are presented in Table 4.

As shown in Table 4, Personalized FVD achieves better prediction performance than Unified FVD for both conservative and aggressive driving styles. For the conservative style, MSEa, RMSEv, and RMSEx are reduced by 9.49%, 6.06%, and 6.78%, respectively. For the aggressive style, the three metrics are reduced by 13.23%, 8.99%, and 11.31%, respectively, indicating that personalized modeling provides a more evident prediction advantage for vehicles whose driving behavior deviates from the average pattern. For the standard driving style, the calibrated parameters $\left(\alpha ,\kappa \right)$ of the two models are relatively close, resulting in similar prediction performance. Overall, the comparison demonstrates the effectiveness of driving-style personalization in representing heterogeneous driving behavior and provides more behavior-specific vehicle motion predictions for subsequent risk assessment and adaptive UAV trajectory planning.

### 4.3 Risk quantification validation

#### 4.3.1 Risk distribution

Figure 8 shows the histogram of risk score distribution across the 130 scenarios. The risk scores range from 0 to 1, with a mean of 0.47 and a standard deviation of 0.20, covering a broad risk spectrum from low to relatively high risk. The distribution is overall concentrated in the low-to-medium risk range, with a certain proportion of long-tail distribution on the high-risk side. By risk level, low-risk scenarios $R_{score}<0.3$ ) account for 19.9%, medium-risk scenarios ( ${0.3\leq R}_{score}<0.$ 6) account for 46.3%, and high-risk scenarios ( $R_{score}\geq 0.$ 6) account for 33.8%. Medium-risk scenarios make up the largest proportion, which is consistent with the typical operating condition of UAVs flying over medium-to-high-density traffic flow during inspection missions in urban environments.

**FIGURE 8:**
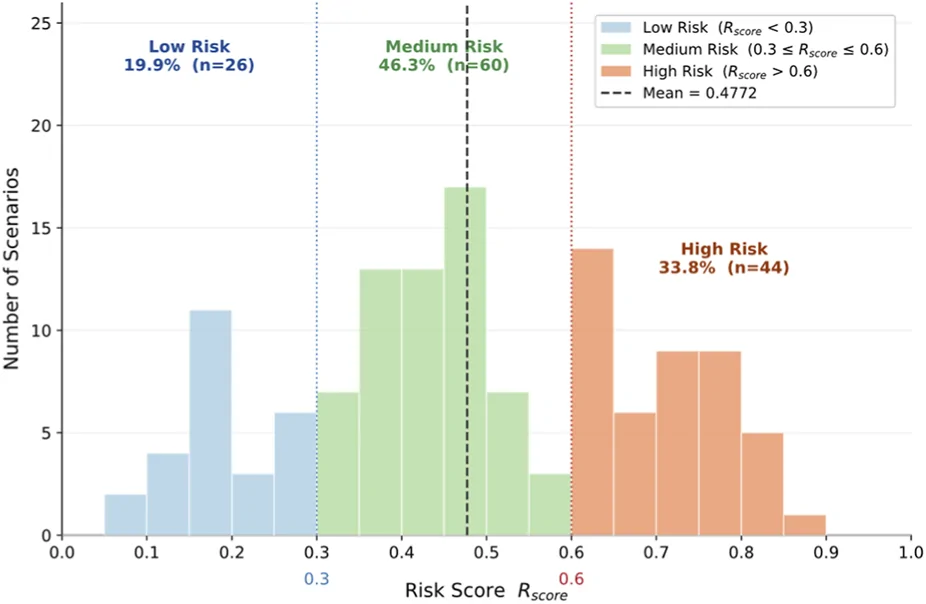
Histogram of risk scores $R_{score}$ .

#### 4.3.2 Risk quantification effectiveness

To objectively evaluate the consistency between the risk scores generated by the fuzzy logic system and the actual safety situation, this study adopts the three-dimensional air-ground time $TTC_{3D}$ to collision as an objective safety reference indicator. $TTC_{3D}$ is defined as the time required for the UAV and the nearest ground traffic participant to reach their closest point in three-dimensional space, under the assumption that the current motion state remains unchanged. It jointly accounts for the horizontal distance $d_{horizontal}$ , flight altitude $h_{vertical}$ , and relative speed $\Delta v_{rel}$ , as shown in Equation 29.

$$TTC_{3D}=\frac{\sqrt{d_{horizontal}^{2}+d_{vertical}^{2}}}{\left\|\Delta v_{rel}\right\|}$$ (29)

A smaller $TTC_{3D}$ indicates a more imminent air-ground conflict and a lower safety margin. A correlation analysis is conducted between the risk score and $TTC_{3D}^{-1}$ to verify the consistency between the output of the fuzzy logic system and the objective safety indicator, with the results shown in Figure 9. The scatter plot on the left shows a clear positive correlation, where scenarios with higher risk scores correspond to larger $TTC_{3D}^{-1}$ values, that is, more imminent air-ground conflicts, with the data points distributed along a linear fitted trend line. The plot on the right in Figure 9 further compares the distribution of $TTC_{3D}^{-1}$ across the three risk levels. The mean value is 0.466 s^-1^ for low-risk scenarios, 0.692 s^-1^ for medium-risk scenarios, and 0.955 s^-1^ for high-risk scenarios. The mean values increase monotonically with risk level, which is highly consistent with the physical understanding of risk perception.

**FIGURE 9:**
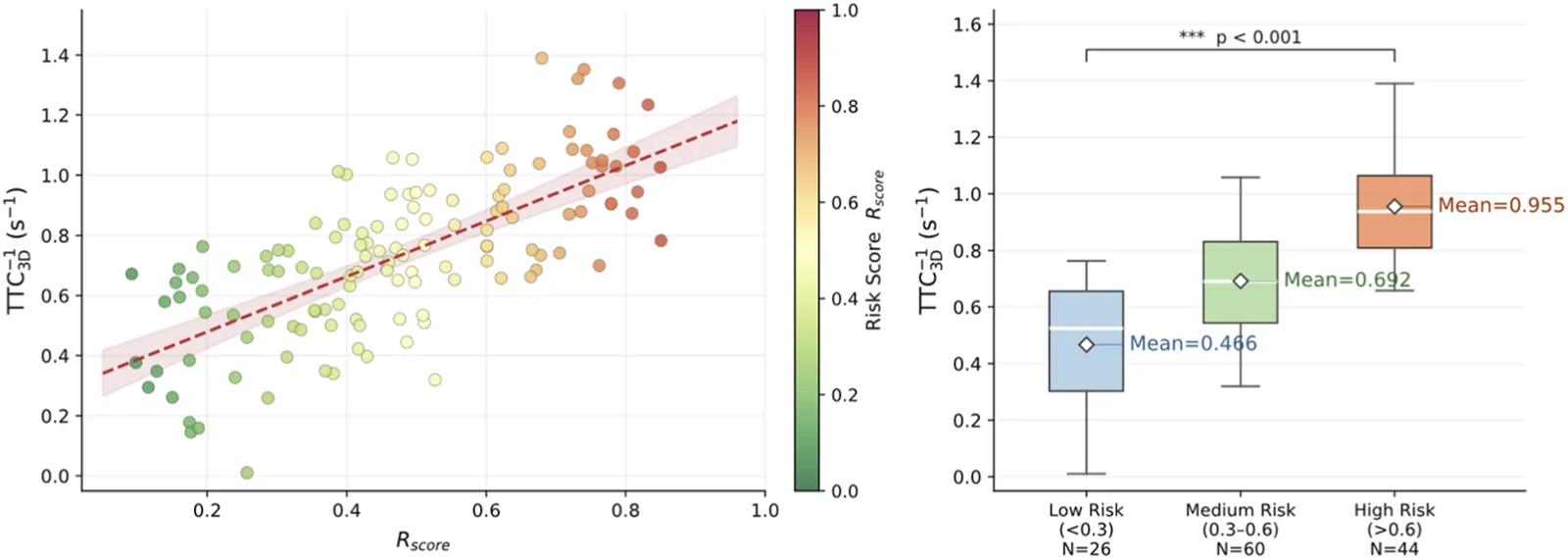
Scatter plot and boxplot of $TTC_{3D}$ quantification.

### 4.4 MS-DGST-VNet validation

#### 4.4.1 Model comparison and ablation study

This section evaluates the effectiveness of the main components of MS-DGST-VNet through ablation experiments by comparing LSTM-only, LSTM + Road-MLP, and the complete MS-DGST-VNet. LSTM-only uses only the historical motion information of the agent for temporal encoding. LSTM + Road-MLP further incorporates road-node features and uses an MLP for feature encoding and fusion. MS-DGST-VNet additionally employs a graph interaction module to model the spatial relationships between agent and road nodes. To ensure a fair comparison, all three models use the same training settings, with the maximum number of training epochs set to 1000. The root mean square errors (RMSEs) of the maneuver time ΔT and three-dimensional displacement parameters, ΔX , ΔY and ΔZ are used as evaluation metrics. The results are shown in Table 5.

**TABLE 5:** Trajectory prediction performance under different model architectures.

Model	RMSE- ΔT (s)	RMSE- ΔX (m)	RMSE- ΔY (m)	RMSE- ΔZ (m)
LSTM-only	0.2693	3.2028	0.3005	2.2647
LSTM + Road-MLP	0.1231	1.4564	0.1806	0.8045
MS-DGST-VNet (ours)	0.1111	1.4557	0.1400	0.7141

As shown in Table 5, LSTM-only exhibits relatively large overall prediction errors. After incorporating road features, LSTM + Road-MLP achieves clear improvements across all four metrics, indicating that road environment information provides useful spatial information for UAV maneuver prediction. By further introducing node-level graph interaction, MS-DGST-VNet reduces the RMSEs of ΔT , ΔY and ΔZ to 0.1111 s, 0.1400 m, and 0.7141 m, respectively. Compared with LSTM + Road-MLP, the prediction errors of ΔY and ΔZ are further reduced by approximately 22.5% and 11.2%, respectively, while the prediction accuracy of ΔX remains nearly unchanged. These results indicate that incorporating road features significantly improves maneuver parameter prediction, while further modeling the interactions between agent and road nodes enhances the representation of spatial relationships in air-ground scenarios.

To further analyze the convergence characteristics of different models, Figure 10 shows the training and validation losses of the three models. LSTM-only converges relatively slowly, with an increasing gap between the training and validation losses in the later training stages. After incorporating road features, LSTM + Road-MLP shows improved convergence. MS-DGST-VNet achieves a faster loss reduction while maintaining a lower validation error. These results indicate that road information and node-level interaction modeling can effectively improve model convergence and generalization.

**FIGURE 10:**
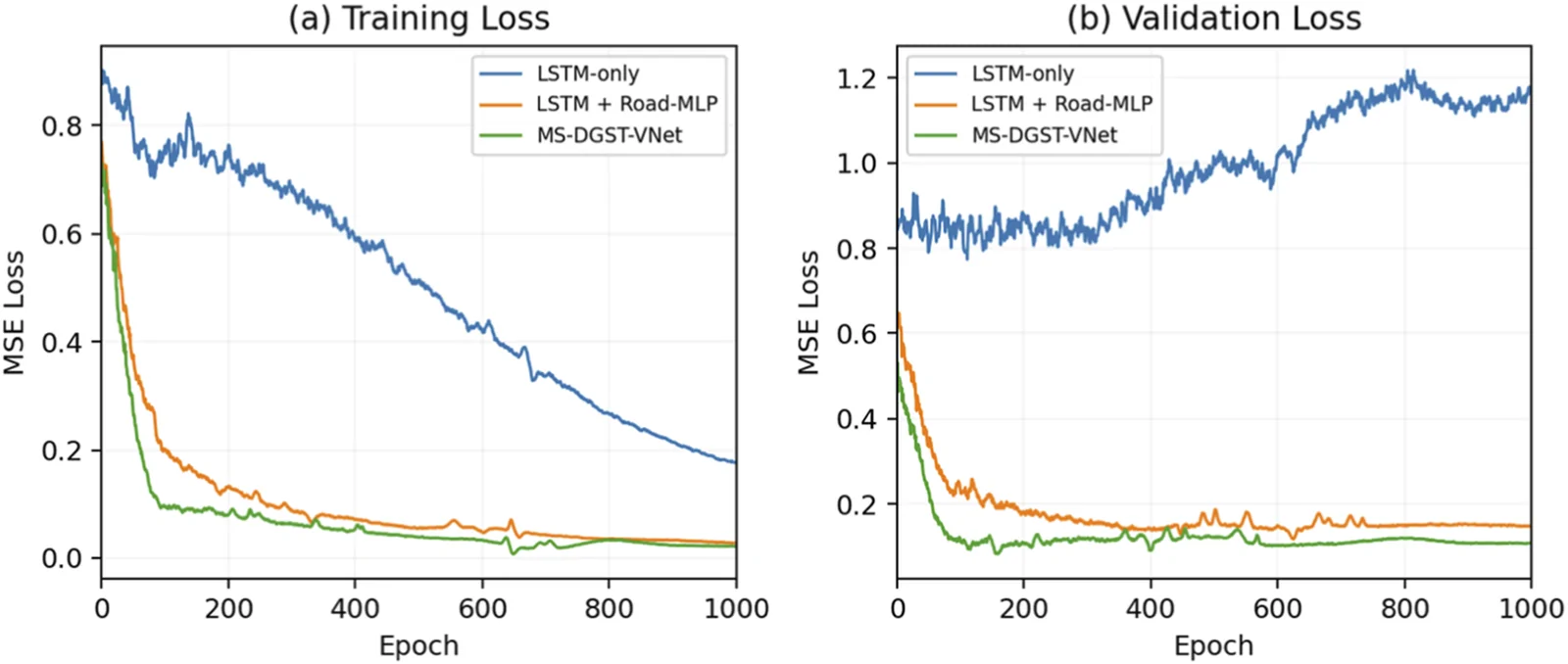
Model loss convergence curves. **(a)** Training Loss, **(b)** Validation Loss.

#### 4.4.2 Encoder depth sensitivity

To analyze the effect of the temporal encoder architecture on model performance, Transformer and LSTM encoders with 1, 3, and 5 layers are compared while keeping the remaining network architecture and training settings unchanged. Similarly, the RMSEs of the maneuver time and three-dimensional displacement parameters, ΔT , ΔX , ΔY and ΔZ , are used to evaluate the prediction performance. The results are shown in Table 6.

**TABLE 6:** Trajectory prediction performance under different encoder configurations.

Encoder	RMSE- ΔT (s)	RMSE- ΔX (m)	RMSE- ΔY (m)	RMSE- ΔZ (m)
Transformer	0.1102	1.7407	0.1346	0.5971
LSTM-1	0.1072	1.5079	0.1430	0.5758
LSTM-3	0.1065	1.6840	0.1396	0.5744
LSTM-5 (ours)	0.1111	1.4557	0.1400	0.7141

As shown in Table 6, different encoders exhibit varying performance across the maneuver parameters. Although Transformer achieves lower errors for some metrics, it shows a relatively large error in longitudinal displacement prediction. In contrast, LSTM is more suitable for capturing the temporal dependencies of continuous motion states through recurrent state updates. Among the LSTM configurations, the five-layer LSTM achieves the lowest RMSE for ΔX while maintaining comparable performance on the other parameters. Therefore, considering the overall prediction performance and temporal representation capability, a five-layer stacked LSTM is adopted as the agent encoder.

### 4.5 Risk-adaptive trajectory planning experiments

#### 4.5.1 Overall performance comparison

To evaluate the UAV trajectory planning performance of the proposed framework, quantitative comparison experiments are conducted on 130 constructed air-ground interaction scenarios. According to the predefined risk classification criteria, the test scenarios are divided into low-risk (R < 0.3), medium-risk (0.3 ≤ R < 0.6), and high-risk (R ≥ 0.6) groups. The planning performance is evaluated separately for each risk level and for all scenarios.

Three methods are compared: (1) Proposed framework, which uses the Personalized FVD model to predict future trajectories of ground vehicles and dynamically adjusts the safety, efficiency, and altitude weights in the trajectory planning cost function according to the real-time risk; (2) Risk-blind planning, which retains the Personalized FVD model and the same trajectory planning cost function but removes the dynamic adjustment of planning weights according to the real-time risk. Specifically, based on the average risk level of the 130 scenarios (R = 0.47), Equations 25–27 yield fixed weights about safety $w_{safe}$ = 4.155, efficiency $w_{eff}$ = 0.092, and altitude $w_{alt}$ = 1.442, which are applied to all test scenarios; and (3) Unified-FVD framework, which uses unified FVD parameters for ground-vehicle trajectory prediction, while keeping the remaining risk assessment and trajectory planning modules identical to those of the proposed framework.

According to the risk-adaptive trajectory planning objective function, the safety cost $J_{safe}$ , efficiency cost $J_{eff}$ , and altitude cost $J_{alt}$ are selected as evaluation metrics. $J_{safe}$ evaluates the safety of the planned UAV trajectory relative to the predicted trajectories of ground vehicles, $J_{eff}$ represents the time cost required for the UAV to complete the maneuver, and $J_{alt}$ characterizes the deviation from the cruising altitude during trajectory planning. The comparison results under different risk levels are shown in Table 7.

**TABLE 7:** Comparison of trajectory planning performance.

Risk level	Method	$J_{safe}$	$J_{eff}$	$J_{alt}$
Low	Proposed framework (ours)	0.0015	8.0913	0.0223
	Risk-blind planning	0.0014	8.3298	0.2519
	Unified-FVD framework	0.0157	8.0671	0.0222
Medium	Proposed framework(ours)	1.4383	8.5286	8.3163
	Risk-blind planning	1.4591	8.5599	8.3689
	Unified-FVD framework	1.6654	8.5317	8.2881
High	Proposed framework(ours)	0.3067	9.0011	51.0175
	Risk-blind planning	0.8246	8.8798	41.9515
	Unified-FVD framework	0.4723	8.9884	50.8291
Overall	Proposed framework(ours)	0.7679	8.6011	21.1102
	Risk-blind planning	0.9528	8.6221	18.1119
	Unified-FVD framework	0.9316	8.5933	21.0334

As shown in Table 7, compared with the Unified-FVD framework, the proposed framework achieves lower $J_{safe}$ in both medium- and high-risk scenarios, with the average value over all 130 scenarios decreasing from 0.9316 to 0.7679, while only minor changes are observed in $J_{eff}$ and $J_{alt}$ .These results indicate that personalized driving-style prediction not only improves ground-vehicle trajectory prediction accuracy but also benefits subsequent risk perception and trajectory planning, thereby improving the safety performance of UAV planning in complex air-ground interaction scenarios.

Compared with Risk-blind planning, the proposed framework exhibits a clearer adaptive trade-off across different risk levels. In low-risk scenarios, $J_{eff}$ decreases from 8.3298 to 8.0913 and $J_{alt}$ decreases from 0.2519 to 0.0223, indicating that dynamic weighting reduces unnecessary conservative maneuvers under low-risk conditions. In high-risk scenarios, $J_{safe}$ decreases from 0.8246 to 0.3067, corresponding to a reduction of approximately 62.81%, while $J_{eff}$ increases only slightly from 8.8798 to 9.0011. These results demonstrate that the risk-adaptive weighting mechanism can dynamically adjust the planning priorities according to the scene risk, achieving improved safety under high-risk conditions through a moderate trade-off in efficiency and increased altitude adjustment.

#### 4.5.2 Case studies

1. Case 1. Low-risk scenario

Scenario description. A UAV performs a road infrastructure inspection mission, flying near the centerline of Lane two at a cruising altitude of 90 m and a cruising speed of 14 m/s. The ground traffic flow beneath consists of six conservative-style vehicles, with two vehicles distributed in each lane, traveling at speeds of approximately 21–23 m/s. The overall traffic density is low, with each vehicle maintaining ample following distance and only small lateral oscillations within its lane.

The planning and simulation results are shown in Figure 11. As shown in Figure 11a, the UAV flies along the direction of Lane 2’s centerline, with its lateral displacement showing small natural fluctuations around the centerline, consistent with the typical flight trajectory of a routine inspection mission. The six conservative ground vehicles travel at approximately 21–23 m/s, faster than the UAV’s cruising speed, and gradually catch up to and overtake the UAV. Figure 11b shows the flight altitude and scenario risk score under the two planning methods. As the ground vehicles approach and overtake the UAV, the dynamic risk score first increases and then decreases, remaining within the low-risk range throughout the mission, with a mean of 0.187 and a maximum of 0.217. No significant altitude avoidance response is triggered, and the proposed framework maintains a flight altitude close to 90 m. In contrast, Risk-blind planning uses R = 0.47 as the fixed reference risk and exhibits slightly greater altitude adjustment under the same scenario, with its altitude trajectory generally higher than that of the proposed framework.

**FIGURE 11:**
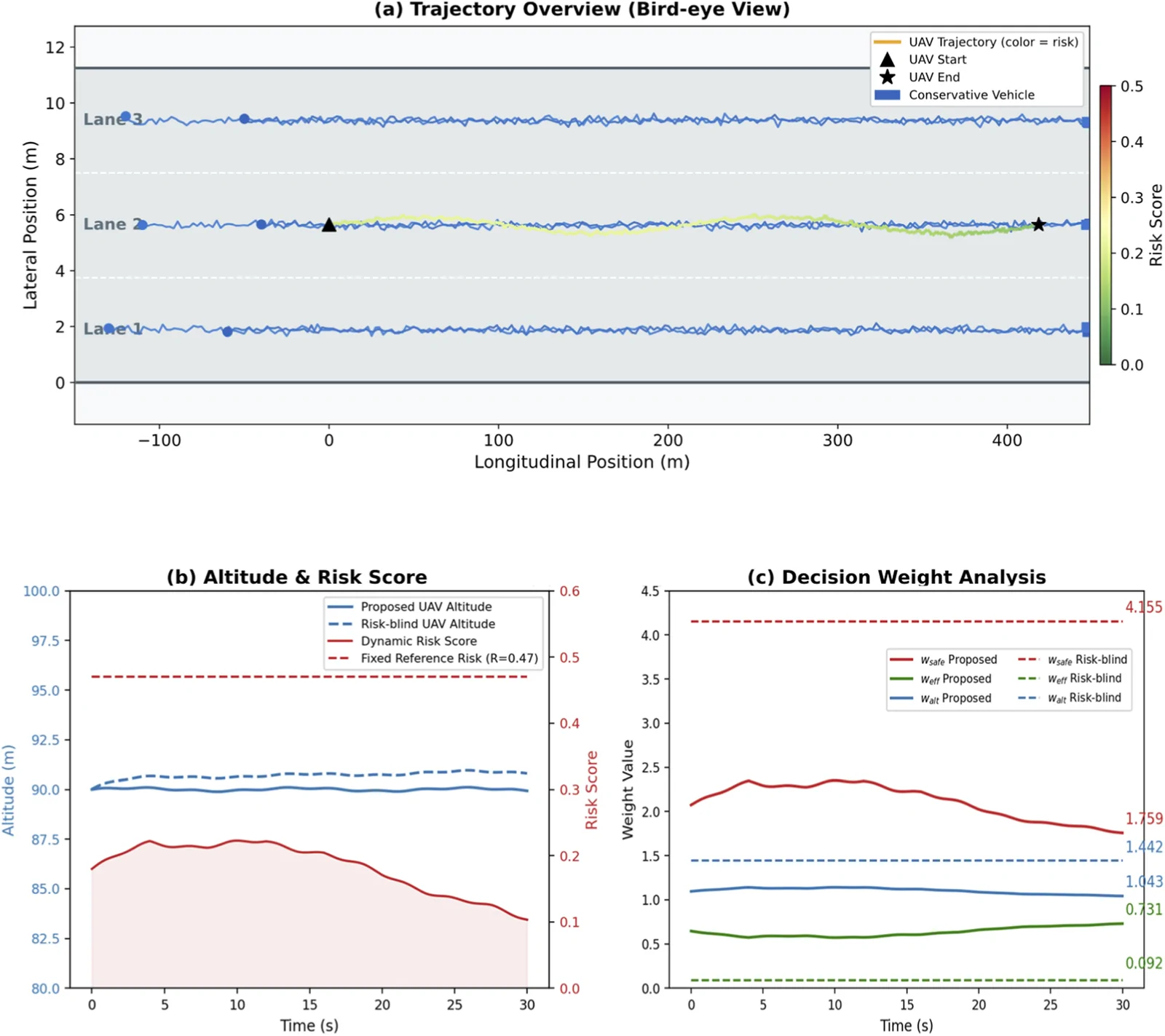
Simulation results of Case 1. **(a)** Trajectory Overview (Bird-eye View), **(b)** Altitude & Risk Score, **(c)** Decision Weight Analysis.

Figure 11c further shows the decision weights of the two methods. The proposed framework dynamically adjusts the safety, efficiency, and altitude weights according to the real-time risk, whereas Risk-blind planning maintains the fixed weights determined by R = 0.47 ( $w_{safe}$ = 4.155, $w_{eff}$ = 0.092 and $w_{alt}$ = 1.442). Since the actual risk in this case is substantially lower than the fixed reference risk, Risk-blind planning assigns a relatively higher safety weight and a lower efficiency weight, resulting in more conservative planning behavior. These results indicate that the risk-adaptive weighting mechanism can adjust the planning preference according to the actual scene risk, thereby reducing unnecessary conservative responses under low-risk conditions.

2. Case 2. High-risk scenario

Scenario description. A UAV performs a dynamic traffic monitoring mission above the road, with an initial cruising altitude of 90 m and a cruising speed of 14 m/s. The ground traffic flow consists of six vehicles with mixed driving styles. Lane 1 contains one conservative and one aggressive vehicle, Lane two contains one standard and one aggressive vehicle, and Lane 3 contains one conservative and one standard vehicle. The conservative vehicles travel strictly along their lane centerlines with minimal lateral offset. The standard vehicles travel along their lane centerlines but exhibit noticeable lateral drift, with relatively large fluctuations in their driving trajectories. The two aggressive vehicles perform lane-changing maneuvers at t = 10 s and t = 15 s, respectively, traveling at a speed of approximately 28 m/s.

The planning and simulation results are shown in Figure 12. As shown in Figure 12a, the UAV performs the monitoring mission above Lane 2, while the three driving styles exhibit distinct motion characteristics. The conservative vehicles maintain relatively stable trajectories, the standard vehicles show noticeable lateral fluctuations within their lanes, and the aggressive vehicles exhibit cross-lane trajectories during lane-changing maneuvers, resulting in stronger dynamic air-ground interactions in the high-risk scenario.

**FIGURE 12:**
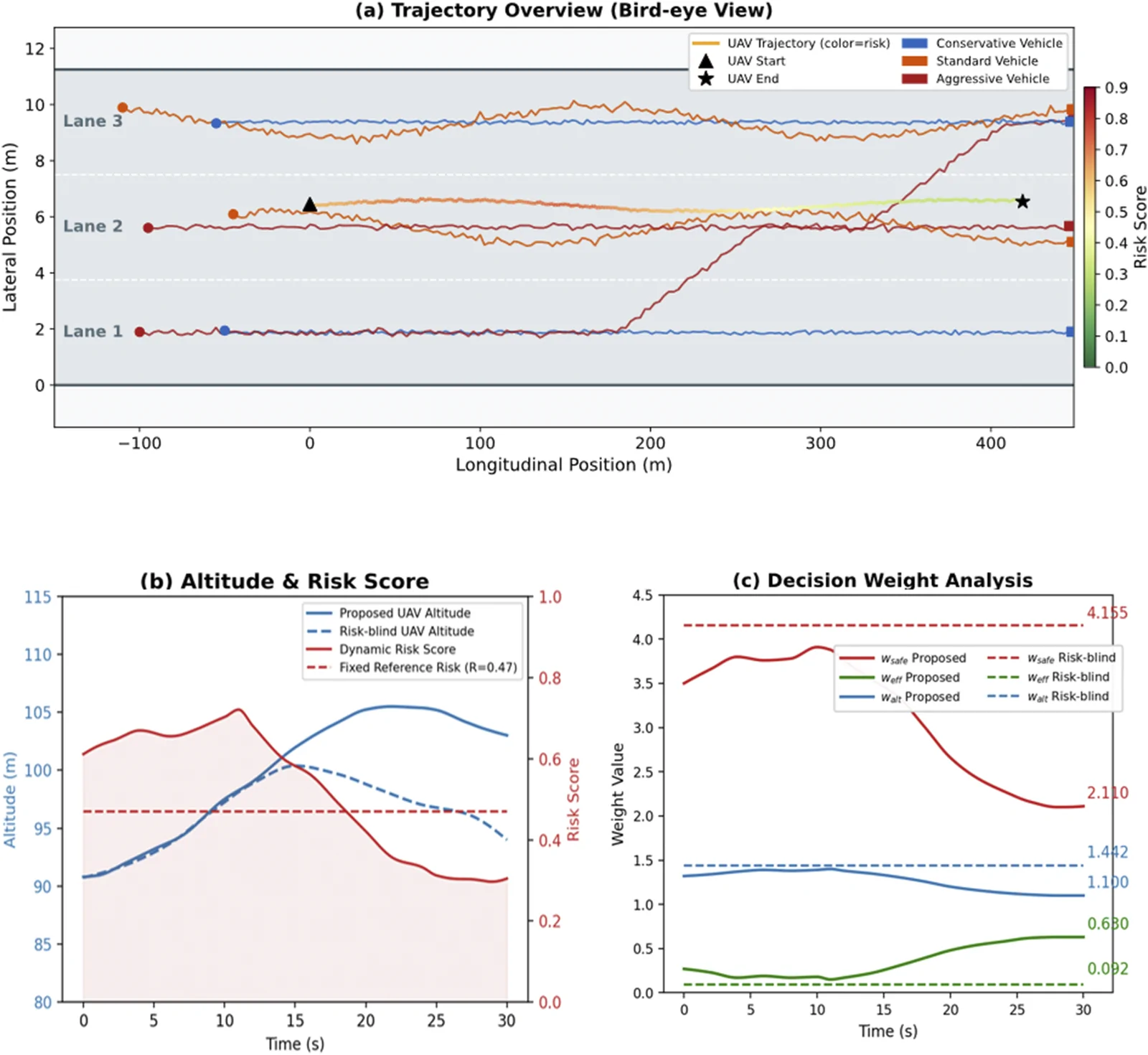
Simulation results of Case 2. **(a)** Trajectory Overview (Bird-eye View), **(b)** Altitude & Risk Score, **(c)** Decision Weight Analysis.

Figure 12b shows the time series of UAV flight altitude and scenario risk score. In the early stage of the mission, the dynamic risk score remains relatively high due to factors such as high vehicle speeds, aggressive lane-changing maneuvers, and short horizontal air-ground distances. The risk reaches its peak at approximately 11 s and then gradually decreases as the relative positions between the vehicles and the UAV change. The proposed framework continuously adjusts the flight altitude according to the dynamic risk, with the UAV gradually climbing from approximately 90 m to a maximum altitude of approximately 105.5 m at t≈ 21.8 s. In contrast, Risk-blind planning, which uses a fixed reference risk of R = 0.47, exhibits a weaker altitude response, reaching a peak altitude of approximately 100.4 m at around 15 s before gradually descending. The difference in altitude response between the two methods becomes more evident during the sustained high-risk period.

Figure 12c further shows the decision weights of the two methods. The $w_{safe}$ , $w_{eff}$ and $w_{alt}$ of Risk-blind planning remain constant throughout the mission, whereas the proposed framework dynamically adjusts the three weights according to the real-time risk. As the scene risk gradually decreases from a high level, $w_{safe}$ decreases accordingly, $w_{eff}$ gradually recovers, and $w_{alt}$ is adjusted simultaneously. Combined with the altitude responses in Figure 12b, the dynamic weighting mechanism enables the UAV to maintain a stronger and more sustained altitude response during the high-risk period, demonstrating the adaptive planning capability of the proposed framework under time-varying air-ground interaction risks.

## 5 Conclusion

To address UAV trajectory planning in heterogeneous air-ground traffic environments, this study proposes an adaptive trajectory planning framework based on dynamic heterogeneity-aware risk perception. The framework predicts the future motion states of ground vehicles through driving style identification and personalized FVD models, quantifies air-ground interaction risk using fuzzy inference and a dynamic risk field, and employs MS-DGST-VNet to predict UAV maneuver parameters for 3D trajectory planning through a risk-adaptive cost function. By establishing a coupled relationship among ground-vehicle behavioral heterogeneity, forward-looking risk perception, and adaptive UAV planning, vehicle behavioral differences can further influence UAV planning decisions through trajectory prediction and risk quantification.

Experimental results based on real-world NGSIM vehicle trajectory data show that the proposed method can effectively characterize vehicle behavioral differences and improve personalized trajectory prediction accuracy. The comparative experiments further demonstrate that personalized prediction, road-environment interaction modeling, and risk-adaptive planning improve the corresponding prediction and planning performance. Finally, the two representative case studies validate that the framework can adaptively adjust its planning strategy according to changes in air-ground interaction risk, achieving a dynamic trade-off between safety and flight efficiency.

The current study mainly focuses on air-ground interactions between a single UAV and ground vehicles in structured road environments, and therefore still has limitations in more complex urban traffic scenarios. Future work will consider different types of traffic participants, such as pedestrians and cyclists, and incorporate 2D/3D multimodal trajectory prediction to characterize non-lane-constrained motion. The framework will also be extended to multi-UAV scenarios by introducing UAV-UAV interactions, conflict constraints, and cooperative planning mechanisms. For different mission types, such as delivery, inspection, and emergency response, mission-specific planning objectives, weights, and constraints can be incorporated to improve the adaptability of the framework to different operational requirements. In addition, the current risk quantification still relies on manually constructed fuzzy rules, which involve a certain degree of prior knowledge. Future work will explore neuro-fuzzy systems or data-driven membership function and rule learning using richer real-world low-altitude interaction data to reduce the subjectivity associated with manual rule design and further improve the adaptability of risk quantification across different scenarios.

## Data Availability

The original contributions presented in the study are included in the article, further inquiries can be directed to the corresponding author.
